# Supplementary material for: Modeling pastoralist movement in response to environmental variables and conflict in Somaliland: Combining agent-based modeling and geospatial data
Source: PLoS One. 2020 Dec 30;15(12):e0244185. doi: 10.1371/journal.pone.0244185 (PMC7773237; doi:10.1371/journal.pone.0244185)
Supplement: S1 Acronyms — (DOCX) [file pone.0244185.s001.docx]

**Supporting Information 1: Acronyms**

ABM: Agent-based model

ACLED: Armed conflict location and event data project

ASALs: Arid and semi-arid lands

FAO: The Food and Agriculture Organization

GIS: Geographic information system

HDX: Humanitarian data exchange

IDPs: Internally displaced populations

KD/KDM: Kernel density/Kernel density map

NIR: Near infrared

NDWI: Normalized difference water index

ODD+D: Overview, design concepts, details, and decisions

RePast: Recursive porous agent simulation toolkit

SAVI: Soil adjusted vegetation index

SDE: Standard deviational ellipse

SWALIM: Somalia Water and Land Information Management

UNFPA: United Nations

UNOCHA: United Nations Office for the Coordination of Humanitarian Affairs

UTM: Universal Transverse Mercator

WGS: World geodetic system
