# Supplementary material for: Modeling pastoralist movement in response to environmental variables and conflict in Somaliland: Combining agent-based modeling and geospatial data
Source: PLoS One. 2020 Dec 30;15(12):e0244185. doi: 10.1371/journal.pone.0244185 (PMC7773237; doi:10.1371/journal.pone.0244185)
Supplement: S1 Data Sources — (DOCX) [file pone.0244185.s002.docx]

# Supporting Information 2: Data Sources

All but one of the data sources used in this research are free and open source. The sources to each dataset can be found below.

## Population distribution

*UNFPA Population Survey Data*

<https://somalia.unfpa.org/sites/default/files/pub-pdf/Population-Estimation-Survey-of-Somalia-PESS-2013-2014.pdf>

## Environment Data

*Administrative boundaries: UN OCHA, obtained from the Humanitarian Data Exchange*

<https://somalia.unfpa.org/sites/default/files/pub-pdf/Population-Estimation-Survey-of-Somalia-PESS-2013-2014.pdf>

*Settlements: UN OCHA Somalia, obtained from the Humanitarian Data Exchange*

<https://data.humdata.org/dataset/somalia-settlements-p-coded-shapefile>

*Slope: DIVA GIS*

<https://www.diva-gis.org/datadown>

*Surface water: NDWI layer from European Union Joint Research Center, obtained from Google Earth*

<https://global-surface-water.appspot.com/>

*Artificial water sources: Somalia Water and Land Information Management (SWALIM)*

Not open source. Obtained through private correspondence with SWALIM. Requests for data can be submitted to [swalim@fao.org](mailto:swalim@fao.org).

*Vegetation data: MODIS Terra Vegetation Indices 16-Day Global*

<https://developers.google.com/earth-engine/datasets/catalog/MODIS_006_MOD13Q1>

*Ethnic boundaries: Kenya Somalia Consortium*

<http://kenyasomali.blogspot.com/2017/07/somali-clans-distribution-in-horn-of.html>

*Conflict data: Armed Conflict Location & Event Data Project (ACLED)*

<https://acleddata.com/data-export-tool/>

*Landcover: Food and Agriculture Organization*

<http://www.fao.org/geonetwork/srv/en/metadata.show?currTab=simple&id=38184>
