# Supplementary material for: Modeling pastoralist movement in response to environmental variables and conflict in Somaliland: Combining agent-based modeling and geospatial data
Source: PLoS One. 2020 Dec 30;15(12):e0244185. doi: 10.1371/journal.pone.0244185 (PMC7773237; doi:10.1371/journal.pone.0244185)
Supplement: S1 File — (DOCX) [file pone.0244185.s003.docx]

**Supporting Information 3: ODD+D Protocol**

The ODD+D protocol documents information used during the modeling process. The ODD protocol (Overview, Design Concepts, and Details) was developed to standardize the documentation of agent-based modeling (Grimm et al, 2010). Müller et al. (2013) suggested the amendment of a section that outlines human decision making, which is relevant to the ABM described in this paper. Therefore, the ODD+D protocol was applied to this study.

1. Overview
   1. **Purpose**
      1. What is the purpose of this study?

The purpose of this study is to understand the impact of seasonal, environmental changes and conflict on the movement of nomadic pastoralists in Somaliland between 2008 and 2018.

- - 1. For whom is the model designed?

This model is designed for humanitarian researchers studying the interaction between conflict, environment, and migration and those studying human migration more generally. This case study also adds to a growing body of literature addressing the value of computer models in the humanitarian sector.

- 1. **Entities, state variables, and scales**
     1. What kind of entities are in the model?

Each modeled agent in the simulation represents a nomadic pastoralist household. The agent remains in a constant state unless the impact of exogenous variables is so severe that the agent state changes from nomadic pastoralist to internally displaced person (IDP).

The second entity in the model is the spatial environment. The environment is not only a modeled entity but also drives the behavior and movement of the agents.

- - 1. By what attributes (i.e. state variables and parameters) are these entities characterised?

The nomadic pastoralists’ clan and ethnicity are identified according to the region in which the agent is generated. The spatial coordinates of the agent’s position are recorded at each timestep in addition to the distance traveled to that location. Lastly, the grid cell value corresponding to the agent’s position is also recorded as an attribute. If the agent state changes from nomadic pastoralist to IDP, the agent effectively exits the simulation and no additional attributes are collected.

The entity representing the spatial environment is composed of aggregated variables in grid cells measuring 1 km². Each grid cell is characterized by a normalized value between 0 and 1. The pixel value reflects the state of the environment as characterized by the following variables: vegetation cover, water availability, terrain gradient, conflict events, and the presence of ethnic boundaries.

- - 1. What are the exogenous factors/drivers of the model?

The drivers of the model are largely captured in the spatial environment as described above. The presence of water and vegetation are considered pull factors whereas conflict, proximity to ethnic boundaries, and steep gradient are incorporated as push factors in the model. Once the agent has identified the most favorable grid cell, it must determine whether they are positions on private or public land. If it is public they can continue their actions, however if the land is private, the agent must establish a deal with the landowner. If the deal is made the agent can remain on that parcel for the duration of a season, if they cannot make a deal then they must continue to search for alternate land. If they are unable to do so within a given season, the agent state changes from pastoralist to IDP and they exit the simulation.

- - 1. If applicable, how is space included in the model?

The modeled environment is a spatial grid, where each grid cell measures 1 km², collectively covering the full extent of Somaliland. The spatial environment is artificially created through the combination of GIS datasets and information derived from satellite imagery. The individual data layers are incorporated into the spatial environment through the following equation:

$$Pixel value = v1 + v2 + v3 - (0.25*v4) - v5 - (0.25*v6)$$

Where,

v1 =normalized vegetative cover, as calculated with SAVI, where L=0.5

v2 = normalized surface water index (enabled in wet seasons)

v3 = artificial water point sources (enabled in dry seasons)

v4 = normalized terrain gradient

v5 = normalized conflict frequency

v6 = ethnic boundary

The additive pixel value is therefore representative of the condition of the artificial environment, which is considered the primary driver of pastoral migration.

- - 1. What are the temporal and spatial resolutions and extents of the model?

The variables are aggregated into a single environmental layer which has a resolution of 1 km² and whose full extent measures 490,000 km². The simulation considers a monthly time step and runs for 11 years between 2008 and 2018.

1. Design Concepts
   1. **Theoretical and empirical background**
      1. What entity does what, and in what order?

Every month, the agent considers a traversable distance in which it searches for a cell with the highest value relative to the cells in the surrounding environment. The cell value reflects the aggregation of environmental variables is composed of favorable variables (high water and vegetation availability) and unfavorable variables (high prevalence of conflict, proximity to ethnic boundaries, or steep terrain. Once the highest grid cell has been identified, the agent moves to this location and is able to stay there for the duration of the season (1 - 3 months).

Once an agent moves to a new grid cell, the agent determines whether they are located on private or public land. If the agent is on public land, they are free to remain at that location without restraints for the duration of a season. If the agent happens to be on private land, the agent must negotiate a land-sharing deal with the local landowner. If the agent successfully strikes a deal with the landowner, they are able to remain at that location for the duration of the season. However, if the agent is unable to obtain access, they are required to move to another grid cell within the same time tick and again consider whether the new grid location is private or public land and repeat the process described above. If the agent moves to grid cells in private land and is also unable to make a deal on three separate occasions in any given season, the agent state switches from pastoralist to IDP, at which point the agent exists the simulation.

- - 1. Which general concepts, theories or hypotheses are underlying the model’s design at the system level or at the level(s) of the submodel(s) (apart from the decision model)? What is the link to complexity and the purpose of the model?

Nomadic pastoralists primarily rely on livestock to support their livelihoods. Pastoralism is therefore deeply intertwined with environmental conditions and is heavily dependent on the availability of water and pasture. While nomadic pastoralists typically traverse familiar environments, unpredictable changes in the landscape have required many to travel longer distances to unfamiliar locations in order to find water and pasture. The changing patterns of movement have at times resulted in ethnic clashes and/or conflict over critical resources. It is therefore the purpose of the model to better understand how conflict and changing environmental variables impact pastoral migration. Through modeling these complex relationships, we hope to develop a better understanding of how agent-based modeling may be used in understanding complex humanitarian crises, and to identify what the gaps and limitations of this methodology are. Of course, this model does not account for all the complexities experienced in reality.

- - 1. On what assumptions is/are the agents’ decision model(s) based?

To our knowledge, there is very limited publicly available data about nomadic pastoralists in Somaliland. The majority of the data that does exist is often outdated. For this reason, this model heavily relies on informed assumptions.

The researchers of this project relied on academic publications, grey literature, and local knowledge to inform agent decision making. The following assumptions were made in this model:

1. SAVI is the indicator used to indicate the presence of vegetation, which is an assumed pull factor for pastoralists
2. Artificial water points and surface water are both used as a pull factor
3. We assumed that the presence of conflict, proximity to ethnic boundaries, and slope in part influence pastoral migration. The likelihood of pastoralists striking a deal with local landowners is assumed, as is the condition for dropping out of pastoralism.
4. Distance scouted and traveled are derived from delphi consensus after conversations with local experts who interact with Somali pastoralist populations. The thresholds utilized in the model are assumptions derived from these consensuses.
   - 1. Why is/are certain decision model(s) chosen?

The agent decision model is largely based on heuristics.

- - 1. If the model/submodel (e.g. the decision model) is based on empirical data, where does the data come from?

Agent decisions do not come from empirical data and primarily rely on heuristics. Data that comprise the environment and come from empirical sources are cited in the Methods section of the article, Table 1.

- - 1. At which level of aggregation were the data available?

The data used to inform agent generation was disaggregated by administrative regions. More granular data at smaller administrative units was largely unavailable.

The variables included in the spatial environment were available at several different spatial resolutions. For instance, the global surface water index was applied to data from Sentinel 2, which captures data at 10 m resolution. The vegetation index was calculated using data from MODIS Terra at 250 m resolution. However, to create one continuous spatial layer, the data layers were aggregated to 1 km resolution. The lower resolution was thought to adequately capture movement on a regional scale and also minimized the time and effort required by the machines running the model.

- - 1. What are the subjects and objects of the decision-making? On which level of aggregation is decision-making modelled? Are multiple levels of decision making included?

Decision-making is modeled on a household level that can then be aggregated to an administrative level.

- - 1. What is the basic rationality behind agent decision-making in the model? Do agents pursue an explicit objective or have other success criteria? How do agents make their decisions?

The basic rationale behind the agent decision-making process is that the agent must identify a grid cell that is suitable for grazing, given a combination of factors. This is done by seeking out the grid cell with the highest additive score in a random proximity to the agent’s location. Once the grid cell has been successfully identified, the agent must confirm that they are able to graze here, since the land may be privately owned. If the agent is consistently able to occupy a grid cell without any problems, they are successful in their mission.

- - 1. Do the agents adapt their behaviour to changing endogenous and exogenous state variables? And if yes, how?

The agent’s decision-making process remains constant, their pattern of movement changes in response to the spatial environment.

- - 1. Do social norms or cultural values play a role in the decision-making process?

Social norms do play a role in the decision-making process. Ethnic conflict is not uncommon in Somaliland, which is why ethnic boundaries are considered in this model. The assumption is therefore, the closer the agent moves to an ethnic boundary, the higher the likelihood for conflict to occur. Additionally, pastoralists are known to strike deals with landowners to gain access to pasture and water as an adaptation technique.

- - 1. Do spatial aspects play a role in the decision process?

Agent decision-making processes and movements are entirely informed by variables that are spatially represented. Distance, terrain gradient, and the general land cover conditions are all considered prior to agent movement. Agents move between 15 - 30 kilometers per timestep, which was determined through discussions captured at a workshop in Nairobi, Kenya on June 3 - 4, 2019.

- - 1. Do temporal aspects play a role in the decision process?

While the agent is able to move at every time tick, the agent is also able to remain at any given grid cell for the duration of a full season, after which they will be required to move. This encourages the agent to follow typical seasonal movement patterns.

- - 1. To which extent and how is uncertainty included in the agents’ decision rules?

Uncertainty is included in the agents’ decision rules through the incorporation of probabilities. Additionally, all the probabilities and even the equation of the additive model can be modified to understand the agents’ behaviors under varied circumstances.

- 1. **Learning**
     1. Is individual learning included in the decision process? How do individuals change their decision rules over time as a consequence of their experience?

Learning is not included in the decision process.

- - 1. Is collective learning implemented in the model?

No

- 1. **Individual sensing**
     1. What endogenous and exogenous state variables are individuals assumed to sense and consider in their decisions? Is the sensing process erroneous?

Individual agents are assumed to sense the surrounding spatial environment (exogenous variable) prior to moving in the simulation.

- - 1. What state variables of which other individuals can an individual perceive? Is the sensing process erroneous?

None.

- - 1. What is the spatial scale of sensing?

The spatial extent of the sensing is determined through a randomized distance between 15 - 30 km.

- - 1. Are the mechanisms by which agents obtain information modelled explicitly, or are individuals simply assumed to know these variables?

The individuals are assumed to know the variables they are sensing.

- - 1. Are the costs for cognition and the costs for gathering information explicitly included in the model?

No

- 1. **Individual prediction**
     1. Which data do the agents use to predict future conditions?

The agent does not predict future conditions.

- - 1. What internal models are agents assumed to use to estimate future conditions or consequences of their decisions?

N/A

- - 1. Might agents be erroneous in the prediction process, and how is it implemented?

N/A

- 1. **Interaction**
     1. Are interactions among agents and entities assumed as direct or indirect?

In this first model iteration, there is no agent interaction.

- 1. **Collectives**
     1. Do the individuals form or belong to aggregation that affect and are affected by the individuals? Are these aggregations imposed by the modeller or do they emerge during the simulation?

A single agent is considered a singular household unit. The agents do not aggregate during the simulation and are affected individually.

- - 1. How are collectives represented?

N/A

- 1. Heterogeneity
     1. Are the agents heterogeneous? If yes, which state variables and/or processes differ between the agents?

The agents are minimally heterogeneous. The agent state variables include geographic position, clan, and ethnic affiliation. Due to limited, outdated, and often unreliable information, no further agent characteristics were included in an effort to minimize assumptions and uncertainty.

- - 1. Are the agents heterogeneous in their decision-making? If yes, which decision models or decision objects differ between the agents?

Agents are homogeneous in their decision-making.

- 1. **Stochasticity**
     1. What process (including initialization) are modelled by assuming they are random or partly random?

The agent start locations are randomly generated with two constraining rules as described in section 2.9.1

- 1. **Observation**
     1. What data are collected from the ABM for testing, understanding and analyzing it, and how and when are they collected?

At every time tick, the following data are collected: 1) geographic position of the agent, 2) the score associated with the pixel within which the agent is situated, and 3) the clan and ethnic affiliation of the agent. The spatial analysis of these attributes will provide insight about where and how far agents move and what the relationship is between agent movement and individual variables.

- - 1. What key results, outputs or characteristics of the model are emerging from the individuals? (Emergence)

We anticipate that varying spatial and temporal patterns of migrations will emerge from the model in response to changing environmental and conflict factors.

1. Details
   1. **Implementation details**
      1. How has the model been implemented?

The model was implemented using Java in Repast.

- - 1. Is the model accessible, and if so where?

The source code is stored on GitHub.

- 1. **Initialization**
     1. What is the initial state of the model world, i.e. at time t=0 of a simulation run?

The state of the model at t = 0 mimics the environment as it was in January 2008. The agents generated at t = 0 are generated randomly throughout Somaliland with two conditions: 1) Agents cannot be in areas that are labeled as water bodies or sand, and 2) agents cannot be within 14 km and 4 km radius from a major city or settlement, respectively.

- - 1. Is the initialization always the same or is it allowed to vary among simulations?

The simulation initialization is always the same.

- - 1. Are the initial values chosen arbitrarily or based on data?

The initial values of the simulation environment are based on aggregated GIS and remotely sensed data that were captured at the time of the simulation date.

The number of agents generated per administrative units are informed by data captured by UNPF in 2013.

- 1. **Input data**
     1. Does the model use input from external sources such as data files or other models to represent processes that change over time?

The spatial environment changes on a seasonal basis, these files were preprocessed by analysts. The input variables and their sources are listed below:

- Agent characteristics | Population distribution is derived from a UNFPA Population Survey conducted in 2014.
- Vegetation | SAVI as calculated on imagery collected by the MODIS Terra satellite
- Conflict | Armed Conflict Location & Event Data Project
- Artificial water sources | Somalia Water and Land Information Management
- Natural water sources | NDWI layer obtained from Google Earth Engine
- Slope | Calculated from DEM obtained from DIVA GIS
- Ethnic boundaries | Obtained from the Kenya Somalia Consortium
- Public/Private land delineation | Point data obtained from HDX, from UNOCHA
