# Supplementary material for: Modeling pastoralist movement in response to environmental variables and conflict in Somaliland: Combining agent-based modeling and geospatial data
Source: PLoS One. 2020 Dec 30;15(12):e0244185. doi: 10.1371/journal.pone.0244185 (PMC7773237; doi:10.1371/journal.pone.0244185)
Supplement: Attachment — Submitted filename: ABM_ResponseToReviewers_09-15-2020.docx [file pone.0244185.s004.docx]

Dear Editors and Reviewers,

We thank you for your thorough review and opportunity to revise and re-submit our manuscript entitled, “Modeling pastoralist movement in response to environmental variables and conflict in Somaliland: Combining agent-based modeling and geospatial data”. We have revised the manuscript based on your reviews and recommendations, which we believe has substantially enhanced the overall manuscript. Below you will find out responses to the editor and reviewer’s comments.

Thank you for considering this resubmission.

Warm regards,

Erica Nelson

On behalf of all co-authors

**Journal requirements:**

1. *Please ensure that your manuscript meets PLOS ONE's style requirements, including those for file naming. The PLOS ONE style templates can be found at*

[*http://www.journals.plos.org/plosone/s/file?id=wjVg/PLOSOne_formatting_sample_main_body.pdf*](https://urldefense.proofpoint.com/v2/url?u=http-3A__www.journals.plos.org_plosone_s_file-3Fid-3DwjVg_PLOSOne-5Fformatting-5Fsample-5Fmain-5Fbody.pdf&d=DwMGaQ&c=WO-RGvefibhHBZq3fL85hQ&r=_rVMzosaL4gZCI-PCgtOu2PeAUgy-dLq6o4VLnPL1eM&m=ift7a-DP_XggngMqCyehIPRjI5DBmT6_Fn8-i65X1JE&s=3v_sLgg4UwwKwSmaEIWgWd8EAWlE51lkMoDKYjmL-Fo&e=) *and* [*http://www.journals.plos.org/plosone/s/file?id=ba62/PLOSOne_formatting_sample_title_authors_affiliations.pdf*](https://urldefense.proofpoint.com/v2/url?u=http-3A__www.journals.plos.org_plosone_s_file-3Fid-3Dba62_PLOSOne-5Fformatting-5Fsample-5Ftitle-5Fauthors-5Faffiliations.pdf&d=DwMGaQ&c=WO-RGvefibhHBZq3fL85hQ&r=_rVMzosaL4gZCI-PCgtOu2PeAUgy-dLq6o4VLnPL1eM&m=ift7a-DP_XggngMqCyehIPRjI5DBmT6_Fn8-i65X1JE&s=IYY5XZQHe6vbmhQv3UAf-2LbTk3VAcHDQKu4ZhwzYVQ&e=)

We have revised the article formatting in accordance to the PLOS ONE’s style requirements as outlined in the included links.

2. *Please provide additional information about the Nairobi workshop for the delineation of private and public land. In particular, please provide additional information about participant inclusion criteria and how consensus was achieved.*

We revised the manuscript at lines 213 – 219 (originally 199 – 206) to address this query. The workshop participants were recommended to the research team by UNICEF Somalia based on job relevance and personal connection to nomadic pastoralists in Somaliland. The participants were either based in Somalia or Somaliland, or were based at a regional office with a focus on Somaliland. Considered key informants with local knowledge, the participant group estimated that approximately 70%- 80% of the land in Somaliland was publicly owned and that private land extended approximately 15 km beyond a large settlement and 5 km beyond smaller towns.

3. *We note that you have stated that you will provide repository information for your data at acceptance. Should your manuscript be accepted for publication, we will hold it until you provide the relevant accession numbers or DOIs necessary to access your data. If you wish to make changes to your Data Availability statement, please describe these changes in your cover letter and we will update your Data Availability statement to reflect the information you provide.*

All but one data source used in this research is free and open source. The links to the original data sources is provided in S2 Supplemental Information.

4. *Thank you for stating the following in the Financial Disclosure section:*

*"This project was funded by the National Center for Civic Innovation Inc (*[*https://www.fcny.org/fcny/about/ncci/*](https://urldefense.proofpoint.com/v2/url?u=https-3A__www.fcny.org_fcny_about_ncci_&d=DwMGaQ&c=WO-RGvefibhHBZq3fL85hQ&r=_rVMzosaL4gZCI-PCgtOu2PeAUgy-dLq6o4VLnPL1eM&m=ift7a-DP_XggngMqCyehIPRjI5DBmT6_Fn8-i65X1JE&s=2HqXOwZnC8LXTY8X4GshAivcTylNi0mrFQnsgPa02J8&e=)*) acting through and on behalf of The Governance Lab (“GovLab”;* [*https://www.thegovlab.org/*](https://urldefense.proofpoint.com/v2/url?u=https-3A__www.thegovlab.org_&d=DwMGaQ&c=WO-RGvefibhHBZq3fL85hQ&r=_rVMzosaL4gZCI-PCgtOu2PeAUgy-dLq6o4VLnPL1eM&m=ift7a-DP_XggngMqCyehIPRjI5DBmT6_Fn8-i65X1JE&s=slInBkw_hAbTtSPTXED94SKYwOP6cdDMeTaAURTOrOg&e=)*). The project grant (no available grant #) funded PGG, ELN, and SAK to conduct this research. This project was originally sponsored by the World Bank (*[*https://www.worldbank.org/*](https://urldefense.proofpoint.com/v2/url?u=https-3A__www.worldbank.org_&d=DwMGaQ&c=WO-RGvefibhHBZq3fL85hQ&r=_rVMzosaL4gZCI-PCgtOu2PeAUgy-dLq6o4VLnPL1eM&m=ift7a-DP_XggngMqCyehIPRjI5DBmT6_Fn8-i65X1JE&s=MzpVEiWuE4UguMuDvLhScBdmEeDnPPVw-_vsr15H16k&e=)*).*

*The funders had no role in study design, data collection and analysis, decision to publish, or preparation of the manuscript." We note that you received funding from a commercial source: 'National Center for Civic Innovation Inc'*

*Please provide an amended Competing Interests Statement that explicitly states this commercial funder, along with any other relevant declarations relating to employment, consultancy, patents, products in development, marketed products, etc.*

*Within this Competing Interests Statement, please confirm that this does not alter your adherence to all PLOS ONE policies on sharing data and materials by including the following statement: "This does not alter our adherence to PLOS ONE policies on sharing data and materials.” (as detailed online in our guide for authors* [*http://journals.plos.org/plosone/s/competing-interests*](https://urldefense.proofpoint.com/v2/url?u=http-3A__journals.plos.org_plosone_s_competing-2Dinterests&d=DwMGaQ&c=WO-RGvefibhHBZq3fL85hQ&r=_rVMzosaL4gZCI-PCgtOu2PeAUgy-dLq6o4VLnPL1eM&m=ift7a-DP_XggngMqCyehIPRjI5DBmT6_Fn8-i65X1JE&s=C4y-9-kDGLqEwTyVioAXsbtSfZttFzacF3BTrvp8Vwk&e=)*). If there are restrictions on sharing of data and/or materials, please state these. Please note that we cannot proceed with consideration of your article until this information has been declared.*

*Please include your amended Competing Interests Statement within your cover letter. We will change the online submission form on your behalf.*

*Please know it is PLOS ONE policy for corresponding authors to declare, on behalf of all authors, all potential competing interests for the purposes of transparency. PLOS defines a competing interest as anything that interferes with, or could reasonably be perceived as interfering with, the full and objective presentation, peer review, editorial decision-making, or publication of research or non-research articles submitted to one of the journals. Competing interests can be financial or non-financial, professional, or personal. Competing interests can arise in relationship to an organization or another person. Please follow this link to our website for more details on competing interests:* [*http://journals.plos.org/plosone/s/competing-interests*](https://urldefense.proofpoint.com/v2/url?u=http-3A__journals.plos.org_plosone_s_competing-2Dinterests&d=DwMGaQ&c=WO-RGvefibhHBZq3fL85hQ&r=_rVMzosaL4gZCI-PCgtOu2PeAUgy-dLq6o4VLnPL1eM&m=ift7a-DP_XggngMqCyehIPRjI5DBmT6_Fn8-i65X1JE&s=C4y-9-kDGLqEwTyVioAXsbtSfZttFzacF3BTrvp8Vwk&e=)

The Competing Interest Statement has been amended within the cover letter.

5. *We note that Figures 1, 3, 4 and 5 in your submission contain [map/satellite] images which may be copyrighted. All PLOS content is published under the Creative Commons Attribution License (CC BY 4.0), which means that the manuscript, images, and Supporting Information files will be freely available online, and any third party is permitted to access, download, copy, distribute, and use these materials in any way, even commercially, with proper attribution. For these reasons, we cannot publish previously copyrighted maps or satellite images created using proprietary data, such as Google software (Google Maps, Street View, and Earth). For more information, see our copyright guidelines:* [*http://journals.plos.org/plosone/s/licenses-and-copyright*](https://urldefense.proofpoint.com/v2/url?u=http-3A__journals.plos.org_plosone_s_licenses-2Dand-2Dcopyright&d=DwMGaQ&c=WO-RGvefibhHBZq3fL85hQ&r=_rVMzosaL4gZCI-PCgtOu2PeAUgy-dLq6o4VLnPL1eM&m=ift7a-DP_XggngMqCyehIPRjI5DBmT6_Fn8-i65X1JE&s=TMGMKHo-qKTiivSofklZCfmeBVsU5n2ZzONXJiL_el0&e=)*.*

*We require you to either (1) present written permission from the copyright holder to publish these figures specifically under the CC BY 4.0 license, or (2) remove the figures from your submission:*

*a)	You may seek permission from the original copyright holder of Figures 1, 3, 4 and 5 to publish the content specifically under the CC BY 4.0 license.*

*We recommend that you contact the original copyright holder with the Content Permission Form (*[*http://journals.plos.org/plosone/s/file?id=7c09/content-permission-form.pdf*](https://urldefense.proofpoint.com/v2/url?u=http-3A__journals.plos.org_plosone_s_file-3Fid-3D7c09_content-2Dpermission-2Dform.pdf&d=DwMGaQ&c=WO-RGvefibhHBZq3fL85hQ&r=_rVMzosaL4gZCI-PCgtOu2PeAUgy-dLq6o4VLnPL1eM&m=ift7a-DP_XggngMqCyehIPRjI5DBmT6_Fn8-i65X1JE&s=LYEeivtEMfZXZ3KVbCq1galnNa5e7ivgiWMsST-42YI&e=)*) and the following text:*

*“I request permission for the open-access journal PLOS ONE to publish XXX under the Creative Commons Attribution License (CCAL) CC BY 4.0 (*[*http://creativecommons.org/licenses/by/4.0/*](https://urldefense.proofpoint.com/v2/url?u=http-3A__creativecommons.org_licenses_by_4.0_&d=DwMGaQ&c=WO-RGvefibhHBZq3fL85hQ&r=_rVMzosaL4gZCI-PCgtOu2PeAUgy-dLq6o4VLnPL1eM&m=ift7a-DP_XggngMqCyehIPRjI5DBmT6_Fn8-i65X1JE&s=Zeu6mQkn_pZyadECMKfRYy4bDtxgcE2NgV9WXsJxjC4&e=)*). Please be aware that this license allows unrestricted use and distribution, even commercially, by third parties. Please reply and provide explicit written permission to publish XXX under a CC BY license and complete the attached form.”*

*Please upload the completed Content Permission Form or other proof of granted permissions as an "Other" file with your submission.*

*In the figure caption of the copyrighted figure, please include the following text: “Reprinted from [ref] under a CC BY license, with permission from [name of publisher], original copyright [original copyright year].”*

*b)	If you are unable to obtain permission from the original copyright holder to publish these figures under the CC BY 4.0 license or if the copyright holder’s requirements are incompatible with the CC BY 4.0 license, please either i) remove the figure or ii) supply a replacement figure that complies with the CC BY 4.0 license. Please check copyright information on all replacement figures and update the figure caption with source information. If applicable, please specify in the figure caption text when a figure is similar but not identical to the original image and is therefore for illustrative purposes only.*

*The following resources for replacing copyrighted map figures may be helpful:*

*USGS National Map Viewer (public domain):* [*http://viewer.nationalmap.gov/viewer/*](https://urldefense.proofpoint.com/v2/url?u=http-3A__viewer.nationalmap.gov_viewer_&d=DwMGaQ&c=WO-RGvefibhHBZq3fL85hQ&r=_rVMzosaL4gZCI-PCgtOu2PeAUgy-dLq6o4VLnPL1eM&m=ift7a-DP_XggngMqCyehIPRjI5DBmT6_Fn8-i65X1JE&s=0RzPHq-2wlAbYEwYb3em5TRnCpcpWteXn5neXw0NeDE&e=)

*The Gateway to Astronaut Photography of Earth (public domain):* [*http://eol.jsc.nasa.gov/sseop/clickmap/*](https://urldefense.proofpoint.com/v2/url?u=http-3A__eol.jsc.nasa.gov_sseop_clickmap_&d=DwMGaQ&c=WO-RGvefibhHBZq3fL85hQ&r=_rVMzosaL4gZCI-PCgtOu2PeAUgy-dLq6o4VLnPL1eM&m=ift7a-DP_XggngMqCyehIPRjI5DBmT6_Fn8-i65X1JE&s=FALnb80s_0mi46GPb50Ntn4o14upNvpgz4LNnizaLMk&e=)

*Maps at the CIA (public domain):* [*https://www.cia.gov/library/publications/the-world-factbook/index.html*](https://urldefense.proofpoint.com/v2/url?u=https-3A__www.cia.gov_library_publications_the-2Dworld-2Dfactbook_index.html&d=DwMGaQ&c=WO-RGvefibhHBZq3fL85hQ&r=_rVMzosaL4gZCI-PCgtOu2PeAUgy-dLq6o4VLnPL1eM&m=ift7a-DP_XggngMqCyehIPRjI5DBmT6_Fn8-i65X1JE&s=m8Q8Mu3uj-3NOiaUFyljewUclWQY29Hos2zJ3QM9h4Q&e=) *and* [*https://www.cia.gov/library/publications/cia-maps-publications/index.html*](https://urldefense.proofpoint.com/v2/url?u=https-3A__www.cia.gov_library_publications_cia-2Dmaps-2Dpublications_index.html&d=DwMGaQ&c=WO-RGvefibhHBZq3fL85hQ&r=_rVMzosaL4gZCI-PCgtOu2PeAUgy-dLq6o4VLnPL1eM&m=ift7a-DP_XggngMqCyehIPRjI5DBmT6_Fn8-i65X1JE&s=RhwosWk1PzXTIiXG9t5ijsdios8I9B5mmmx4h695rJI&e=)

*NASA Earth Observatory (public domain):* [*http://earthobservatory.nasa.gov/*](https://urldefense.proofpoint.com/v2/url?u=http-3A__earthobservatory.nasa.gov_&d=DwMGaQ&c=WO-RGvefibhHBZq3fL85hQ&r=_rVMzosaL4gZCI-PCgtOu2PeAUgy-dLq6o4VLnPL1eM&m=ift7a-DP_XggngMqCyehIPRjI5DBmT6_Fn8-i65X1JE&s=-OTi49-2ekD500hpStN1pLUt9UYpk9H3MowwjOIGedc&e=)

*Landsat:* [*http://landsat.visibleearth.nasa.gov/*](https://urldefense.proofpoint.com/v2/url?u=http-3A__landsat.visibleearth.nasa.gov_&d=DwMGaQ&c=WO-RGvefibhHBZq3fL85hQ&r=_rVMzosaL4gZCI-PCgtOu2PeAUgy-dLq6o4VLnPL1eM&m=ift7a-DP_XggngMqCyehIPRjI5DBmT6_Fn8-i65X1JE&s=KfJAIwfbkCD0dn-dZM-bUROy5t0M4HrcdwqpSHI58ls&e=)

*USGS EROS (Earth Resources Observatory and Science (EROS) Center) (public domain):* [*http://eros.usgs.gov/#*](https://urldefense.proofpoint.com/v2/url?u=http-3A__eros.usgs.gov_-23&d=DwMGaQ&c=WO-RGvefibhHBZq3fL85hQ&r=_rVMzosaL4gZCI-PCgtOu2PeAUgy-dLq6o4VLnPL1eM&m=ift7a-DP_XggngMqCyehIPRjI5DBmT6_Fn8-i65X1JE&s=JTpYATLo_dxIDvOrRBt_hVVZBc4wHV3gNwBHHDp05Wk&e=)

*Natural Earth (public domain):* [*http://www.naturalearthdata.com/*](https://urldefense.proofpoint.com/v2/url?u=http-3A__www.naturalearthdata.com_&d=DwMGaQ&c=WO-RGvefibhHBZq3fL85hQ&r=_rVMzosaL4gZCI-PCgtOu2PeAUgy-dLq6o4VLnPL1eM&m=ift7a-DP_XggngMqCyehIPRjI5DBmT6_Fn8-i65X1JE&s=1uBBNAt97lheO93SaYMquQUkfdzu_aMUyvPqnzKlG30&e=)

Thank you for bringing this to our attention for clarification. The figures were created using open source data and were modified to include the copyright information in the figure.

**Additional Editor Comments:**

*I concur with the recommendations of both reviewers. The revised version of the paper must clearly address methodological issues identified by both reviewers. In addition to reporting model calibration and validation results, global sensitivity analysis is highly recommended as well. Novel insights from the proposed ABM and its generalizability potential to simulate other pastoral societies must also be addressed in the discussion section.*

Thank you for your valuable feedback. We admit that our limited computing capacity hindered this research, but fortunately we were recently able to gain access to Harvard University’s supercomputer cluster. Access to the cluster enabled multiple iterations of the model with different population sizes (10%, 25%, 75%) and multiple iterations holding the populations size constant (10%). Cross comparison of the outcomes of these runs provided internal validation of the model. By prioritizing the generation of additional simulations for internal model verification, we unfortunately did not have time to focus on model calibration and validation.

We have further added language to the limitations section of the paper to address the uncertainty of fixed and variable parameters relative to a global sensitivity analysis and its utility for this model. Given the multi-disciplinarity of the data and the narrow ranges of the variables, we provide a justification for our logic about not running a GSA.

Additionally, in the discussion section, we added additional language to comment on the model generalizability and scalability, particularly its utility as a potential tool for the global humanitarian community whose interest in migration triggering factors and migration dynamics ultimately inform their work. Well versed in conflict factors, organizations in this sector are only just beginning to understand the environmental and climate science influencing population movement but lack the capacity for analysis that modeling offers.

*[Note: HTML markup is below. Please do not edit.]*

***Reviewers' comments:***

*Reviewer's Responses to Questions*

***Comments to the Author***

*1. Is the manuscript technically sound, and do the data support the conclusions?*

*The manuscript must describe a technically sound piece of scientific research with data that supports the conclusions. Experiments must have been conducted rigorously, with appropriate controls, replication, and sample sizes. The conclusions must be drawn appropriately based on the data presented.*

*Reviewer #1: Partly*

*Reviewer #2: Partly*

*2. Has the statistical analysis been performed appropriately and rigorously?*

*Reviewer #1: N/A*

*Reviewer #2: No*

*3. Have the authors made all data underlying the findings in their manuscript fully available?*

*The* [*PLOS Data policy*](https://urldefense.proofpoint.com/v2/url?u=http-3A__www.plosone.org_static_policies.action-23sharing&d=DwMGaQ&c=WO-RGvefibhHBZq3fL85hQ&r=_rVMzosaL4gZCI-PCgtOu2PeAUgy-dLq6o4VLnPL1eM&m=ift7a-DP_XggngMqCyehIPRjI5DBmT6_Fn8-i65X1JE&s=h8N_7nXTJlUq2RIn95Q2OMLoq1UT8OFM-KGDHdvotmA&e=) *requires authors to make all data underlying the findings described in their manuscript fully available without restriction, with rare exception (please refer to the Data Availability Statement in the manuscript PDF file). The data should be provided as part of the manuscript or its supporting information, or deposited to a public repository. For example, in addition to summary statistics, the data points behind means, medians and variance measures should be available. If there are restrictions on publicly sharing data—e.g. participant privacy or use of data from a third party—those must be specified.*

*Reviewer #1: Yes*

*Reviewer #2: Yes*

*4. Is the manuscript presented in an intelligible fashion and written in standard English?*

*PLOS ONE does not copy edit accepted manuscripts, so the language in submitted articles must be clear, correct, and unambiguous. Any typographical or grammatical errors should be corrected at revision, so please note any specific errors here.*

*Reviewer #1: Yes*

*Reviewer #2: Yes*

*5. Review Comments to the Author*

*Please use the space provided to explain your answers to the questions above. You may also include additional comments for the author, including concerns about dual publication, research ethics, or publication ethics. (Please upload your review as an attachment if it exceeds 20,000 characters)*

*Reviewer #1: Summary: The authors present an ABM of pastoralist mobility in Somaliland, designed to assess the impacts of environmental variability and conflict on pastoralist movement and the potential for pastoralist drop-out due to loss of herds. This is an important topic for reasons that the authors describe, and I think that the authors frame the problem appropriately and provide a clear presentation of their work. As there is a need for more published descriptions of ABMs on this topic I would like to see the paper published. That said, I have several comments on formulation and experimental design that I would like to see addressed prior to publication. It appears that the authors were severely limited by computing capacity in this study, and for this reason I'm not sure that they can address my concerns with model and simulation design. The "right" solution to that problem is for the authors to get access to more computing power; this is a big ABM, but it's not historically big, and assuming it is coded in a reasonable fashion it should be possible to do more complete simulations and analysis of output given reasonable computing resources. If this is not possible then I don't intend to block publication, for the reasons stated above, but these limitations really do handicap the value of the study.*

*General comments:*

*1. It appears that the only two time-varying drivers of migration are SAVI and conflict data. The nature of their temporal variability is not presented in the results, but it should be. This could be as simple as district-averaged SAVI and district total # of conflicts by year and season, 2008-2018. This would allow the reader to see trends and variability relevant to the authors' hypotheses, and would allow the authors to speak to these proposed drivers in a more concrete way. A time series of the average favorability score by district would also be helpful.*

Thank you for this recommendation. We have included a table in the results section that captures average SAVI value per administrative region per season and total conflict per administrative region for each season for the duration of the study period to enable temporal comparison to the changing number of agents per month per administrative region.

*2. There's no way for the reader to understand the relative importance of conflict and environment based on the results as they are presented. One way to deal with this would be to repeat the simulation but with interannual environmental variability eliminated (i.e., climatology), which in this case I think would mean calculating favorability using climatologically averaged SAVI. The same could be done with a constant conflict rate. Doing this would require performing additional simulations, but since it does not require any increase in computational demand over the baseline model I would hope that the authors are able to do it.*

Thank you for this wonderful recommendation. Unfortunately, due to limited time and resources we were unable to generate additional simulation results considering constant variables. However, we did generate additional simulation results considering a random sample of 10%, 25%, and 75% of the agent population. Additionally, we have developed tables capturing the temporal variability of SAVI, conflict events, and population numbers for every time step during the simulation using a 10% agent sample. We elaborate on these graphs in the Results and Discussion sections of the paper.

*3. I appreciate that computing limitations can be a problem, but it seems very odd to me that the authors are only able to show 2008 and 2018 output. This limitation really drives down the interpretability of the study and the richness of its results. I strongly encourage the authors, if it is at all possible, to find a computing solution that will allow them to look at output from every year in the simulation. Without this the results are really quite thin, and they can't be applied to extract an understanding of conflict and environment impacts on ABM dynamics in any robust way.*

We apologize that this was not clearly communicated in the manuscript. The simulation generated results on a monthly basis for the entire period between January 2008 to December 2018, but the spatial analyses consider the beginning and ending years to compare overall movement across space and time. We have taken the time to generate additional simulations and have expanded our analysis to include information across more timepoints in the last section of the results.

*Specific comments:*

*line 132-133: I appreciate the need to develop models for Somaliland, but in what other ways does the research in this paper differ from the models listed above in this paragraph? The paper would be more compelling if the ABM work were framed in terms of innovation (or at least alternative formulation) relative to existing ABM of pastoralism in East Africa.*

Thank you for this important point. We added language to clarify that the novel aspect of our model is to include empirical conflict data, SAVI instead of NDVI, the use of a different variable/agent interaction model (an aggregated favorability surface), and the utilization of spatial analysis to characterize spatial trends and generate associative hypotheses. The combination of the variables and the application of the model to Somaliland and Puntland are additionally unique in existing literature. This work builds on progress made by previous modeling projects considering East African populations. We are guided by this previous work and have incorporated elements we felt would be critical considerations to humanitarian stakeholders.

*line 174: What is the source of the NDWI data? Google Earth is a platform, not a primary data source. Some information on these data is required, since the authors later assume that all mapped waterbodies are indicative of rainy season water extent in the study period (with both rainy seasons having the same water coverage(?)), and that water coverage is negligible in the dry season.*

The source of the NDWI data is the European Union Joint Data Center. To the authors’ knowledge, there is no dataset that captures sparse surface water coverage of water bodies in Somaliland. Given data limitations, this dataset was used to represent both rainy seasons. The water coverage is negligible in the dry season according to literature and the workshop participants and was therefore excluded as a dataset in the dry seasons.

*line 291-295: This is a critical rule, and I don't quite understand the logic. Why wouldn't a pastoralist choose to move to public land, even if it is not the most favorable grid cell, after negotiations fail, rather than letting their herd die and becoming an IDP?*

The model logic used by this simulation requires the agent to seek out the most favorable grid cell score. The score is considered the most important consideration and the distinction between private and public land is not as important. This decision making process was subjectively embedded into the model and could be considered a limitation.

*line 343 et seq.: How does this approach account for the possibility that agents interact with each other? It would seem that simulating only 10% of households would make interactions--namely, competition for a favorable grid cell--less common, such that behaviors and resulting spatial distributions in a simulation that included 100% of households might be systematically different from a simulation that includes only 10% of households. Some explanation on this point would be useful.*

The simulation does not account for agent interaction. However, in response to this excellent point, we generated additional simulations that consider a random sample of 50% and 75% of the agent population to consider how these samples would manifest differently in comparison to a 10% sample. We found that the sample thresholds produced similar results and therefore generated additional simulations considering 10% of the population to capture the variability in random sampling. These results have been appended in the manuscript.

*line 495: "given the assumption that..." Is this an assumption or a known aspect of pastoralism in this area? I appreciate that there is little data on pastoral activities in this region, but surely there must be at least narrative accounts of seasonal migration patterns?*

Long-distance migration is considered one of several coping mechanisms for pastoralists. Citations have been added to reflect this knowledge.

*line 555: I don't understand why it's not possible to look at model output at times other than 2008 and 2018. This strikes me as a huge limitation, since it means the authors can't look at whether the climatic events that they hypothesize to be drivers of change actually correspond to times and locations of significant population change. I appreciate that this is a big model, but is it really impossible to store output? I would expect that an ABM on this scale could be run on a reasonably powerful server or cloud service in a way that would allow the operator to store output.*

We apologize that this was not clearly communicated. The model simulates on a monthly basis from January 2008 to December 2018. The data outputs capture monthly agent positions in this time period, but the analysis presented in this paper largely considered the beginning and end years. In the revisions we have included analysis on additional timepoints.

*Reviewer #2: My biggest concern about the manuscript is the missing of validation. Even though empirical validation is difficult due to data paucity, it is still necessary to carry out some theoretical validations, that is to use the model to simulate some well-known and/or widely accepted theories. Without validation, the exercise is just a baseless numerical game.*

*Additionally, the spatial analysis techniques are quite rudimentary and do not provide much insights. The necessary sensitivity analysis for such models is also missing. It is rather disappointing that the model does not discover any thresholds, non-linearity, emergence, or other phenomena of complexity that make the ABM worthwhile.*

*Overall, I think the model needs to be fundamentally improved in order to produce scientifically valuable results.*

The lack of external validation is a fair criticism given the multiple data inputs and the uncertainty of agent decision-making in particular. Our goal in developing the model, was to invite an opportunity for real-world validation, particularly amongst academics like ourselves who work closely with multiple tiers (global to local) humanitarian actors and multi-lateral agencies. It is our intent to investigate face validity of the model with this group of stakeholders in the future.

Given the fact that is model is a proof-of-concept, preliminary model that can simply not currently be externally validated given the paucity of data at this time, the generation of thresholds seems unnecessary given the fact that they would be so inaccurate when superimposed upon real-world phenomena. Other behaviours are now further explored with the addition of spatio-temporal population, conflict, and SAVI trends. However, we agree that now that the larger trends have been characterized, future, more nuanced analysis can be undertaken.

In regards to internal validity, the narrative now reflects the multiple simulation runs using a range of sample sizes resulting in low variance (<1.2% for all iterations). To rightly address the issue of global sensitivity analysis et al., we added the following narrative to the Limitations section:

“As in many complex models, inputs have a degree of uncertainty and require the significant computational methods of sensitivity analyses, model calibration, and validation, to efficiently understand real-world non-linear scenarios. Although we did not quantify uncertainty for each of the model’s variable parameters due to time and resource constraints, the parameter values are not wide and thus physically constrained, and with the exceptions of the environmental indices, are mostly binary, all of which theoretically limit the degree of uncertainty in the model. Notably, four of the model’s variables—slope, ethnic boundaries, artificial water sources, and land ownership—are fixed. That said, the lack of a sensitivity analysis for the remaining variable parameters– either local or global – limits the predictability of the model.

Our goal for the model was to explore how environmental, conflict, and land tenure variables can impact pastoralist livelihood and migrations. While this model is limited by a variety of factors, including data availability and resources, this research is intended to provide an exploratory window into the interactions of these domains in data-scarce environments. We also intend to inspire and inform additional thinking about next generation modelling that can be even more robust, more specific, and more precise with quantifiable uncertainties. For instance, a global sensitivity analysis, often used to quantify uncertainty in environmental impact science, neural networks, and climate change effects, might be useful in a multi-disciplinary model such as ours to gain added confidence for targeting interventions in migratory populations.”

*6. PLOS authors have the option to publish the peer review history of their article (*[*what does this mean?*](https://urldefense.proofpoint.com/v2/url?u=https-3A__journals.plos.org_plosone_s_editorial-2Dand-2Dpeer-2Dreview-2Dprocess-23loc-2Dpeer-2Dreview-2Dhistory&d=DwMGaQ&c=WO-RGvefibhHBZq3fL85hQ&r=_rVMzosaL4gZCI-PCgtOu2PeAUgy-dLq6o4VLnPL1eM&m=ift7a-DP_XggngMqCyehIPRjI5DBmT6_Fn8-i65X1JE&s=f7cqrr5xU_daVA4wr2CAz-K-qsTPOiY9nYJzufuX3ME&e=)*). If published, this will include your full peer review and any attached files.*

*If you choose “no”, your identity will remain anonymous but your review may still be made public.*

***Do you want your identity to be public for this peer review?*** *For information about this choice, including consent withdrawal, please see our* [*Privacy Policy*](https://urldefense.proofpoint.com/v2/url?u=https-3A__www.plos.org_privacy-2Dpolicy&d=DwMGaQ&c=WO-RGvefibhHBZq3fL85hQ&r=_rVMzosaL4gZCI-PCgtOu2PeAUgy-dLq6o4VLnPL1eM&m=ift7a-DP_XggngMqCyehIPRjI5DBmT6_Fn8-i65X1JE&s=fc90A4sQJnTNXkV37AhpjzUPq-cfjdh2-YnUBgiSlNM&e=)*.*

*Reviewer #1: No*

*Reviewer #2: No*

*[NOTE: If reviewer comments were submitted as an attachment file, they will be attached to this email and accessible via the submission site. Please log into your account, locate the manuscript record, and check for the action link "View Attachments". If this link does not appear, there are no attachment files to be viewed.]*

*While revising your submission, please upload your figure files to the Preflight Analysis and Conversion Engine (PACE) digital diagnostic tool,* [*https://pacev2.apexcovantage.com/*](https://urldefense.proofpoint.com/v2/url?u=https-3A__pacev2.apexcovantage.com_&d=DwMGaQ&c=WO-RGvefibhHBZq3fL85hQ&r=_rVMzosaL4gZCI-PCgtOu2PeAUgy-dLq6o4VLnPL1eM&m=ift7a-DP_XggngMqCyehIPRjI5DBmT6_Fn8-i65X1JE&s=L-vuuu66cnGAtGUq-gtlPZTl9c-BEA4qu0JsMjAcNdA&e=)*. PACE helps ensure that figures meet PLOS requirements. To use PACE, you must first register as a user. Registration is free. Then, login and navigate to the UPLOAD tab, where you will find detailed instructions on how to use the tool. If you encounter any issues or have any questions when using PACE, please email us at figures@plos.org. Please note that Supporting Information files do not need this step.*

We have uploaded our figures through PACE.
